# Changes in Intra-to-Extra-Cellular Water Ratio and Bioelectrical Parameters from Day-Before to Day-Of Competition in Bodybuilders: A Pilot Study

**DOI:** 10.3390/sports10020023

**Published:** 2022-02-14

**Authors:** João Pedro Nunes, João P. M. Araújo, Alex S. Ribeiro, Francesco Campa, Brad J. Schoenfeld, Edilson S. Cyrino, Michele C. C. Trindade

**Affiliations:** 1 Metabolism, Nutrition, and Exercise Laboratory, Physical Education and Sport Center, Londrina State University, Londrina 86057-970, Brazil; edilsoncyrino@gmail.com; 2 Department of Physical Education, State University of Maringá, Maringa 87020-900, Brazil; araujo.jpma@gmail.com (J.P.M.A.); trindade.michele@yahoo.com.br (M.C.C.T.); 3 Center for Research in Health Sciences, University UNOPAR-Pitágoras, Londrina 86047-790, Brazil; alex.sribeiro@kroton.com.br; 4 Department for Life Quality Studies, University of Bologna, 47921 Rimini, Italy; francesco.campa3@unibo.it; 5 Health Sciences Department, CUNY Lehman College, Bronx, NY 10468, USA; bradschoenfeldphd@gmail.com

**Keywords:** body composition, bodybuilding, resistance training, carbohydrate loading, peaking, dehydration, bioelectrical impedance vector analysis

## Abstract

The present study analyzed the effects from day-before to day-of bodybuilding competition on intracellular water (ICW), extracellular water (ECW), total body water (TBW), and bioimpedance analysis (BIA) parameters (resistance, R; reactance, Xc; and derived scores) in bodybuilding athletes. We assessed anthropometry and BIA (foot-to-hand; tetrapolar; 50 kHz) in 11 male bodybuilders (29 ± 4 year-old; 81 ± 8 kg; 172 ± 7 cm; 27 ± 2 kg/m^2^) both on the pre-competition day and on the contest day. Results revealed significant increases in ICW (31.6 ± 2.9 to 33.1 ± 2.8 L), with concomitant decreases in ECW (19.8 ± 1.8 to 17.2 ± 1.4 L) and TBW (51.4 ± 4.6 to 50.3 ± 4.2 L) from the day-before competition to contest day, which resulted in relatively large increases in the ICW/ECW ratio (1.60 ± 0.03 to 1.92 ± 0.01 L). Moreover, significant increases in R (391 ± 34 to 413 ± 33 ohm), Xc (64 ± 7 to 70 ± 6 ohm), and phase angle (9.3 ± 0.6 to 9.6 ± 0.7 degree) were observed between time periods. The phase angle scores reported on show-day of 9.6 and 11.2 appear to be the highest group mean and individual values observed in the literature to date. In conclusion, the strategies carried out on the final day of peak-week bodybuilding preparation lead to changes in BIA parameters and body water, with fluids shifting from the extra- to the intracellular compartment.

## 1. Introduction

Bodybuilding athletes are judged on muscular aesthetics. Successful competitors must present a combination of high muscle volume, symmetry and proportion, and very low levels of body fat [1,2]. These objectives require the implementation of a diverse array of strategies to optimize physique aesthetics on show-day. Preparation for bodybuilding competition generally involves two phases: an off-season phase, in which the primary goal is to optimize muscle hypertrophy; and a pre-contest phase, in which the focus is to reduce subcutaneous body fat as low as possible while simultaneously maintaining muscle mass [1,2,3,4,5].

At the end of the pre-contest phase, usually beginning the prior week to show-day (the “peak” week), athletes often adopt specific strategies that differ from the off-season and pre-contest phases [2]. The primary objective of the peak week is to maximize muscular appearance, definition, and tightness (i.e., the skin is pulled firmly against muscle with no signs of subcutaneous water). During peak week, bodybuilders commonly employ a carb-deplete/carb-load strategy, whereby carbohydrate intake is reduced during the first few days of the peak week (thereby reducing muscle glycogen levels) and then consumed in higher amounts in the days leading up to show-day (thereby causing a supercompensation of muscle glycogen storage) [2,6]. In parallel, daily water intake is increased to about four times greater than normal intake during the week, and then reduced in the day-before show [2,7,8]. This strategy is posited to cause polyuria in the days approaching the contest, thus reducing total body water (TBW) [2,6,8]. Moreover, the supercompensation of glycogen theoretically causes an osmotic effect that pulls the subcutaneous extracellular water (ECW) into the muscles [8], maximizing muscle volume on contest day by increasing the intracellular water (ICW) [8,9,10,11].

Although such peaking strategies are based on sound physiological rationales, they lack sufficient empirical evidence to guide practical application [8,12]. A few studies have endeavored to explore the effects of peak-week refeeding on increasing muscle thickness in bodybuilders [6,13,14]. For example, de Moraes [6] analyzed 24 male bodybuilders during the end of the peak week. The authors reported that only the athletes that performed carbohydrate loading increased arm muscle thickness, and limb circumferences and body mass. Moreover, improvements in muscular aesthetics in a photo silhouette evaluation were observed only in those who followed such a peaking strategy [6]. However, despite speculation that an increase in muscle size due to carbohydrate refeeding might indicate an increase in intracellular water (ICW) [10,11], the effect on ECW is not known, nor is it known whether an ECW-to-ICW shift occurs.

In addition to the possible fluid shift from the extracellular to intracellular compartments, a loss of TBW may occur near the competition, especially in bodybuilders of weight-limit classes, who seek to cut weight rapidly and report the use of diuretic substances [2,8]. However, the use of a reference method for body water evaluation (i.e., the isotope dilution technique) is not always practically feasible. As an alternative, bioelectrical impedance analysis (BIA) represents an attractive alternative for evaluating body fluids in sport. Foot-to-hand analysis is the most common and reliable way to perform BIA in clinical and research settings [15,16]. With this approach, whole-body water can be estimated with good agreement compared to reference methods [15,16]. Besides, BIA is a quick, simple, non-invasive, and relatively low-cost technology [15,16]. While the main use of BIA involves the estimation of body composition through predictive equations, the bioelectrical parameters, resistance (R) and reactance (Xc), provide important information as well, especially when assessed through bioelectrical impedance vector analysis (BIVA) [15,16,17,18,19,20]. R is inversely related to TBW content while Xc represents the muscle cell membrane’s integrity, quality, and density [17,18]. BIVA consists of the combined evaluation of R and Xc as a vector within a Cartesian graph [18,21,22], in which the position of the vector is considered in relation to its x/y axes displacement, which is then reflected by the phase angle (PhA), a bioelectrical parameter positively related to the ICW/ECW ratio [15,16,23].

Since a change in body fluids may occur in proximity to the bodybuilding show-day, studies analyzing such parameters could provide important insights for nutritionists and coaches who are advising bodybuilders on nutritional status, hydration, and body composition in preparation for competitions. However, as indicated by a recent systematic review on the topic [2], there is a paucity of studies on the topic, highlighting the need for objective research. To fill gaps in the current literature, the present investigation aimed to evaluate changes in body water fractions and BIA parameters from the day-before to day-of competition in bodybuilders. We hypothesized that TBW and ECW would decrease, ICW would increase, and R and Xc would increase.

## 2. Materials and Methods

### 2.1. Experimental Design

The present study was carried out over two days (day-before and competition-day, on Saturday and Sunday, respectively), in the afternoon period, during a state-level bodybuilding competition from an associated federation of the International Federation of BodyBuilding and Fitness (IFBB). During the day-before, participants were approached after the weigh-in to inquire if they were interested in participating in the study. Following a detailed description of the study procedures, those who agreed to participate completed a written informed consent, and underwent anthropometric and BIA assessments. On show-day, before the warm-up for the first call-outs to stage, participants were re-evaluated on the same variables in an air-conditioned (~22 °C) backstage room. This study was approved by the University Ethics Committee and was conducted according to the Declaration of Helsinki [24].

### 2.2. Subjects

Approximately 50 male and female athletes were invited to take part in the present study on the day prior to the contest. Of this sample, 11 male competitors agreed to participate (age = 28.8 ± 4.1 years [range: 22–35]; weight = 80.5 ± 7.9 kg; stature = 172.0 ± 7.2 cm; body mass index = 27.2 ± 1.9 kg/m^2^; experience = 6 ± 4 competitions). One athlete competed in a junior category (≤23 years-old, ≥75 kg) while 10 competed in weight-limit classes (men’s physique = 5; men’s bodybuilding = 5).

### 2.3. Body Composition

Bodyweight was assessed using a portable digital scale with values obtained to the nearest 0.1 kg (Glass 10, G-Tech; Rio de Janeiro, Brazil). Athletes self-reported their stature based on values obtained at weigh-in by the contest’s organizer. Waist, right-side upper-arm, and mid-thigh circumferences were assessed to the nearest 0.1 cm via an anthropometric tape measure (TR4013, Sanny; São Bernardo do Campo, Brazil). Measurements were performed in triplicate, and the median was taken as the final value. Only 8 participants agreed to have their body circumferences assessed on show-day.

A single frequency phase-sensitive BIA device (BIA/Vitality Analyzer™, The Nutritional Solutions Corporation Ltd.; Harrisville, USA) was used to assess R and Xc bioelectrical parameters, with a frequency of 50 kHz, at 450 μA, and an accuracy of 1.0 ohm [25]. PhA was subsequently calculated as arc-tangent (Xc/R) × 180°/π, and BIVA scores were calculated with the values of R, Xc, and stature [21,22]. Before assessing BIA, subjects were instructed to remove all objects containing metal and assume a supine position for 10 min on gymnastics mats, isolated from the ground and electrical conductors, with legs abducted at 45°, shoulders abducted at 30° relative to the body midline, and hands pronated. In accordance with standard procedures, we cleaned the participant’s skin with alcohol and placed 2 electrodes on the surface of the right hand and 2 on the right foot [25]. In total, 5 foot-to-hand measurements were taken, or up to 3 successive ones, with variance less than 10.0 ohms on R and 3.0 ohms on Xc (i.e., coefficient of variation (CV) <3%), and we used the mean of these values for analysis. Measurements were carried out with athletes fasting for at least 1.5 h. The same experienced researcher performed the assessments on both days.

Body water fractions were estimated using the formulas from Matias et al. [26], based on the gold-standard deuterium/bromide-dilution method in a young lean athletic sample, as follows:

TBW = (0.286) + (0.195 × (S^2^/R)) + (0.385 × Wt) + (5.086 × Sex).
ECW = (1.579) + (0.055 × (S^2^/R)) + (0.127 × Wt) + (0.006 × (S^2^/Xc)) + (0.932 × Sex).
ICW = TBW − ECW.

where Wt is weight in kg, S is stature in cm, R is resistance in ohm, Xc is reactance in ohm, and sex was 1 for men. Matias et al. [26] reported CVs < 1.0% for R and Xc measurements [26].

### 2.4. Statistical Analyses

Paired-sample t-tests were used to compare the effects between day-before and day-of contest assessments. The one-sample Hotelling’s T^2^ test was used to determine if bioelectrical vectors’ changes were significantly different from zero (null vector). A *p* < 0.05 was accepted as statistically significant. Cohen’s d effect size (*d*) was calculated as show-day mean minus day-before mean, divided by day-before standard deviation [27], and was corrected for bias in small samples [28]. An effect size of 0.00–0.19 was considered as trivial, 0.20–0.49 as small, 0.50–0.79 as moderate, and ≥ 0.80 as large [27]. In addition, we ran linear regressions to identify whether age played a role on the magnitude of the results (age as a covariate; day-before—show-day change score as a dependent factor). The data were analyzed using JASP software (Jasp Stats, v.1.0; Amsterdam, Netherlands), and presented as the mean and standard deviation (sd), unless otherwise stated.

## 3. Results

**Figure 1:**
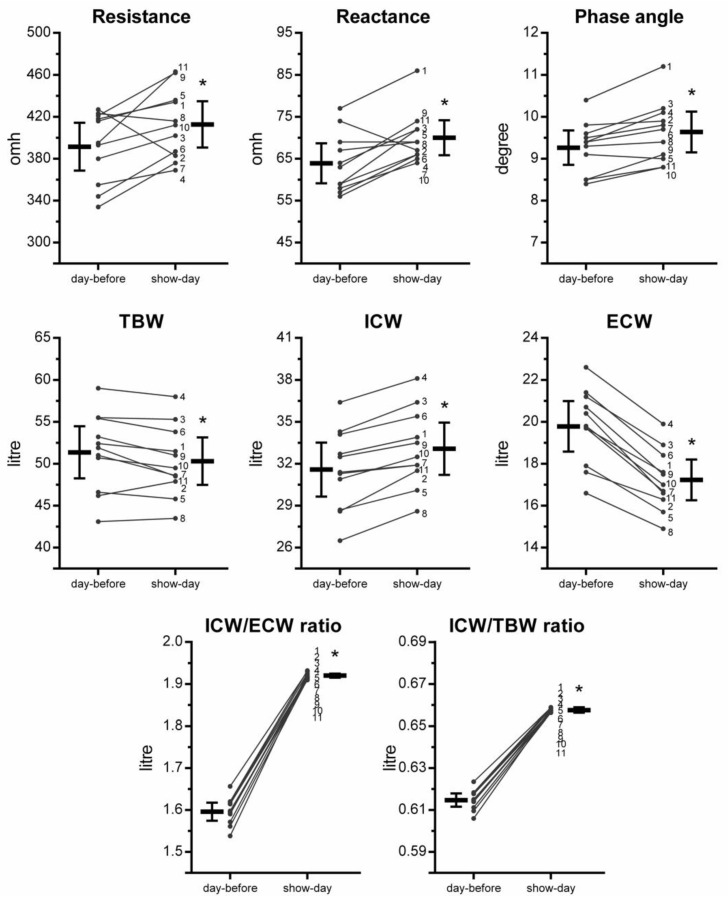
Individual values for BIA-derived scores, and body water measures in bodybuilders from day-before to show-day (*n* = 11). TBW = total body water; ICW = intracellular water; ECW = extracellular water. Data are mean and 95% confidence intervals. * = *p* < 0.05 vs. day-before.

Figure 1 displays the individual values for R, Xc, PhA, TBW, ICW, ECW, ICW/ECW ratio, and ICW/TBW ratio. Significant changes were observed in R (391.5 ± 33.9 omh; 412.7 ± 33.0 omh; observed power [op] = 0.436; *p* = 0.037; *d* = 0.60), Xc (63.9 ± 7.1 omh; 70.0 ± 6.2 omh; op = 0.699; *p* = 0.005; *d* = 0.83), PhA (9.3 ± 0.6 degree; 9.6 ± 0.7 degree; op = 0.412; *p* = 0.001; *d* = 0.58), TBW (51.4 ± 4.6 L; 50.3 ± 4.2 L; op = 0.102; *p* = 0.028; *d* = −0.22), ICW (31.6 ± 2.9 L; 33.1 ± 2.8 L; op = 0.323; *p* < 0.001; *d* = 0.50), ECW (19.8 ± 1.8 L; 17.2 ± 1.4 L; op = 0.985; *p* < 0.001; *d* = −1.39), ICW/ECW ratio (1.60 ± 0.03 L; 1.92 ± 0.01 L; op = 1.000; *p* < 0.001; *d* = 9.77), and ICW/TBW ratio (0.61 ± 0.01 L; 0.66 ± 0.01 L; op = 1.000; *p* < 0.001; *d* = 8.70) but not in bodyweight (80.8 ± 7.9 kg; 80.2 ± 8.0 kg; op = 0.055; *p* = 0.158; *d* = −0.07).

**Figure 2:**
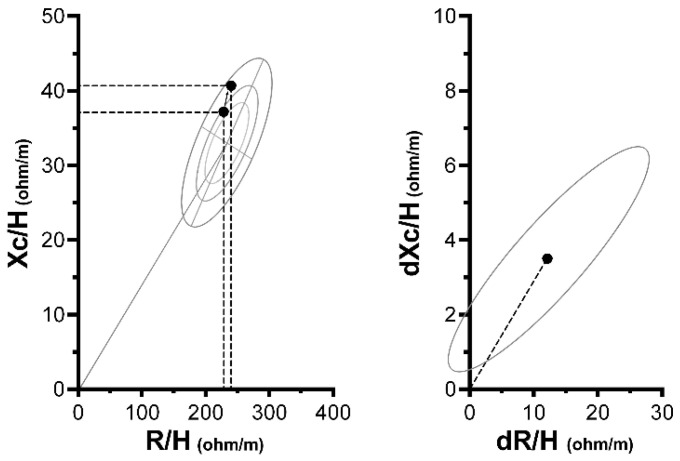
Results for BIVA scores in bodybuilders from day-before to show-day (*n* = 11). R = resistance; Xc = reactance; H = height (stature, in meter). Left panel: R–Xc graph with mean vector displacement, indicated by the black dots (day-before → show-day), plotted on the reference tolerance ellipses for lean male athletes from Campa et al. [29]. Right panel: day-before to show-day mean vector displacement with 95% confidence ellipses (T^2^: *p* < 0.001).

Figure 2 displays the results for BIVA. The simultaneous increase in R/H (228.0 ± 7.9 ohm/m; 240.0 ± 17.5 ohm/m; op = 0.334; *p* = 0.040; *d* = 0.51) and Xc/H scores (37.2 ± 4.7 ohm/m; 40.7 ± 3.3 ohm/m; op = 0.566; *p* = 0.006; *d* = 0.71) resulted in a significant vector displacement from the day-before to show-day (T^2^ = 15.2; F = 7.1; op = 0.850; *p* < 0.001; D^2^ = 1.19). In addition, we observed significant decreases in waist circumference (79.3 ± 3.2 cm; 78.6 ± 2.8 cm; op = 0.097; *p* = 0.036; *d* = −0.21) and increases in upper-arm circumference (36.1 ± 1.5 cm; 36.8 ± 1.5 cm; op = 0.225; *p* = 0.028; *d* = 0.40), whereas no change was observed in mid-thigh circumference (56.6 ± 2.6 cm; 56.2 ± 2.0 cm; op = 0.071; *p* = 0.468; *d* = −0.14). Furthermore, for all variables, linear regression analyses showed that age was not a significant factor in any model (*p* values ranged from 0.156 to 0.926)

## 4. Discussion

The present study showed significant changes in body water fractions and BIA parameters from the day-before competition to show-day in competitive bodybuilders. We observed a large increase in the ICW/ECW ratio, which was mediated by a moderate increase in ICW and a large decrease in ECW. The findings confirm previous hypotheses [2], based on evidence showing improvements in muscle size and body silhouette images [6,13,14], and anecdotal bodybuilding reports [8]. The observed effects were accompanied by a small decrease in TBW and moderate-to-large increases in BIA R, Xc, and PhA. In addition, we observed a small increase in the upper-arm circumference while the waist circumference decreased, and the mid-thigh circumference showed no significant change.

The increases in ICW, ICW/ECW, and ICW/TBW occurred as expected. Interestingly, the positive changes in these variables, and the decreases in ECW, were observed in all the subjects (Figure 1). As noted in the introduction, these effects may be attributed to strategies customarily employed by bodybuilders during the peak week that involve manipulating the diet, particularly carbohydrates and water [2,7,8]. The higher amount of carbohydrate consumed immediately prior to competition conceivably enhances muscle fullness given that each gram of glycogen binds 3–4 g of water [9]. The manipulation of carbohydrates in the diet is especially important given that the reduction in carbohydrate intake for several days followed by overfeeding is thought to increase glycogen stores above normal values [2,30,31,32]. Aiming to reduce TBW and ECW, elevated amounts of water (e.g., 10 L per day [8]) are consumed at the beginning of the peak week to then cause polyuria close to show day [2,8]. The use of diuretic drugs and/or supplements (e.g., vitamin c, herbals) may facilitate the reduction in TBW while the supercompensation of glycogen stores may help to pull water from the extracellular space into the muscles, thereby further reducing ECW [2].

In regard to girth measures, we observed a decrease in the waist circumference, which is considered important for obtaining a “v-taper” of the trunk (reduced waist:upper-trunk size ratio) [2,7]. Although this effect was relatively small and observed only in some of the athletes, it could have a meaningful impact on the competitors’ placings. We also observed a small increase in the circumference of the upper arm. This finding is consistent with that of de Moraes et al. [6], where it was observed that bodybuilders performing carbohydrate loading from day-before to show day showed increased ultrasound-assessed muscle thickness of the elbow flexors and extensors. However, our results showed no significant changes in thigh circumference, and this distinct effect between the upper and lower limbs has also been noted in previous studies [2,6,13]. Although an underlying explanation is not readily apparent, this phenomenon may be due to some athletes avoiding training sessions that produce high amounts of muscle damage in the lower limbs during the peak week [2]. Thus, with less training-induced glycogen depletion of the lower limbs, carbohydrate refeeding might have provided only a minor effect on increasing muscle size in this body region. Studies that directly compare the changes in ICW and ECW in upper vs. lower body segments are needed to obtain clarity on this topic.

Overall, the athletes had an ICW/ECW ratio of 1.6 L on the day-before (ranging from 1.54 to 1.66) and 1.9 L on show day (ranging from 1.91 to 1.93 L). Moreover, the ICW/TBW was 0.61 L on the day-before (ranging from 0.606 to 0.624) and 0.66 L on show day (strictly ranging from 0.656 to 0.659 L). These changes are not only statistically significant and of a relatively large magnitude, but the show-day ratios are higher than those previously reported in lean physically active young adults (~1.41 L; ~0.585 L [33]), lean young athletes (~1.54 L; ~0.608 L [26]), and bodybuilders assessed prior to the peak week (~1.52 L; ~0.602 L [34]; ~1.54 L; ~0.607 L [35]; ~1.66 L; ~0.625 L [33]). In particular, previous studies in bodybuilders did not observe such high values of ICW in relation to ECW and TBW during the other phases of bodybuilding preparation [28,29], even when body water was assessed via the gold-standard deuterium/bromide-dilution method [28]. Thus, it can be speculated that these observed ratios (~1.9 ICW/ECW and ~0.66 ICW/TBW) are transient and refer to the “peak” osmolarity conditions that can be achieved during bodybuilding preparation. Accordingly, in addition to visual inspection of an athlete [2,6], analyzing BIA-estimated body water fractions during the peak week can provide objective insight into strategies for improving body composition up to these mentioned ratios. For example, if ICW/ECW and ICW/TBW ratios are appreciably lower than 1.9 and 0.66 L during the days before the show, athletes may consider adopting “peaking” strategies to improve muscle “fullness” (e.g., carbohydrate loading). Further studies in high-level international competitors are required to determine whether these values can be surpassed.

Regarding the raw BIA values, we observed increases in R, Xc, and PhA. The increase in R can be attributed to a decrease in TBW (i.e., dehydration, especially in the ECW), since these two parameters are inversely correlated [15,23]. Interestingly, contrasting results have been found in athletes after a muscle injury [36], where both R and Xc decreased, reflecting reduced cellular integrity due to cell damage, losses in ICW, and ECW accumulation [15]. In the present study, ECW loss amounted to 2.6 L on average while TBW loss was ~1.1 L, implying a state of dehydration. However, participants increased ICW by ~1.5 L; thus, the ICW/ECW and ICW/TBW ratios increased. This effect was concomitantly reflected in the increased Xc and PhA values, suggesting a fluid shift from the extracellular to the intracellular compartments [15,23]. Regarding the BIVA, the simultaneous increases in R/H and Xc/H generated a vector elongation (increasing mostly along the y axis), which indicates decreases in TBW with increases in ICW/ECW [15,29].

On an individual level, subject n. #1 presented intriguing BIA results (see Figure 1). This athlete displayed the highest observed PhA and Xc values in the two days of evaluation. Moreover, his PhA of 11.2 degrees on show day is 4.4 z-scores greater (in sd units) than the reference mean value for lean male athletes [29], and, seemingly, is the highest PhA value reported to date in the literature [15,17,20,23,29,33,37,38,39,40,41,42]. Interestingly, this participant was the most experienced athlete in terms of participation in competitions (15 contests) within the present sample, has experience in competitions at the international level for another bodybuilding federation, and was the overall category winner at the event in which the present data were assessed. A photo of the athlete on show-day is provided in the Supplementary Material to illustrate the degree of conditioning associated with such a high PhA value. The show-day PhA mean of 9.6 degrees of the present sample also stands out over previously reported PhA values of 3.0–5.0 in sarcopenic adults, 5.0–6.0 in physically active older individuals, 6.0–7.0 in healthy young adults, and 7.0–8.0 in athletes of various sports [15,17,20,23,29,33,37,38,39,40,41,42].

### Limitations, Gaps, and Directions

This study has some limitations that should be noted when attempting to draw practical inferences. First, our sample size was relatively small. Although we attempted to recruit a high number of participants for assessment, the athletes approached in the given competition showed low interest in participating. Second, future studies may consider assessing BIA outcomes throughout the entire peak-week period, and assessing other variables concerning body composition. Third, we performed whole-body BIA. Further segmental assessments with the same foot-to-hand BIA equipment plus measures of body/limb circumferences would provide important insights into the effects of the peaking strategy on each body region [15,16]. Moreover, portable A- or B-mode ultrasound devices are viable options under these circumstances [6,13], providing additional information about muscle and subcutaneous-fat thicknesses. Fourth, we did not obtain information about the training and dietary strategies adopted for each athlete during the peak week. Given the manipulation of these factors may influence the magnitude of the results [2,6,7], future studies could consider analyzing them to determine which peaking strategy (diet and training) elicits optimal results. For example, the amount of training volume carried out and the amount of carbohydrate ingested in the refeeding may impact the effectiveness of the glycogen depletion–supercompensation strategy. Finally, the bodybuilders were not drug tested for the competition. Thus, it is not clear if, and to what extent, the use of anabolic drugs may have influenced the results, limiting the ability to generalize findings to drug-free athletes.

## 5. Conclusions

In conclusion, significant changes in body water fractions and BIA parameters were displayed during the last days of preparation for a bodybuilding competition. We observed increases in R, Xc, PhA, ICW, ICW/ECW, ICW/TBW, and upper-arm circumference, and decreases in TBW, ECW, and waist circumference.

### Practical Applications

Our results suggest that customary anecdotal bodybuilding strategies used from the day-before to show day appear to be effective in promoting positive alterations in the proportion of ICW over ECW and TBW. In this regard, it is suggested that the muscles conceivably were “fuller”, with a reduced level of subcutaneous body water, leading to a more “defined” on-stage appearance.

## Data Availability

The full-data spreadsheet is available in the Supplementary Materials.

## Supplementary Materials

The following supporting information can be downloaded at: https://www.mdpi.com/article/10.3390/sports10020023/s1, Figure S1: Athlete photo, Table S1: Database spreadsheet.
