# Supplementary figures and images for: Changes in Intra-to-Extra-Cellular Water Ratio and Bioelectrical Parameters from Day-Before to Day-Of Competition in Bodybuilders: A Pilot Study
Source: Sports (Basel). 2022 Feb 14;10(2):23. doi: 10.3390/sports10020023 (PMC8880471; doi:10.3390/sports10020023)

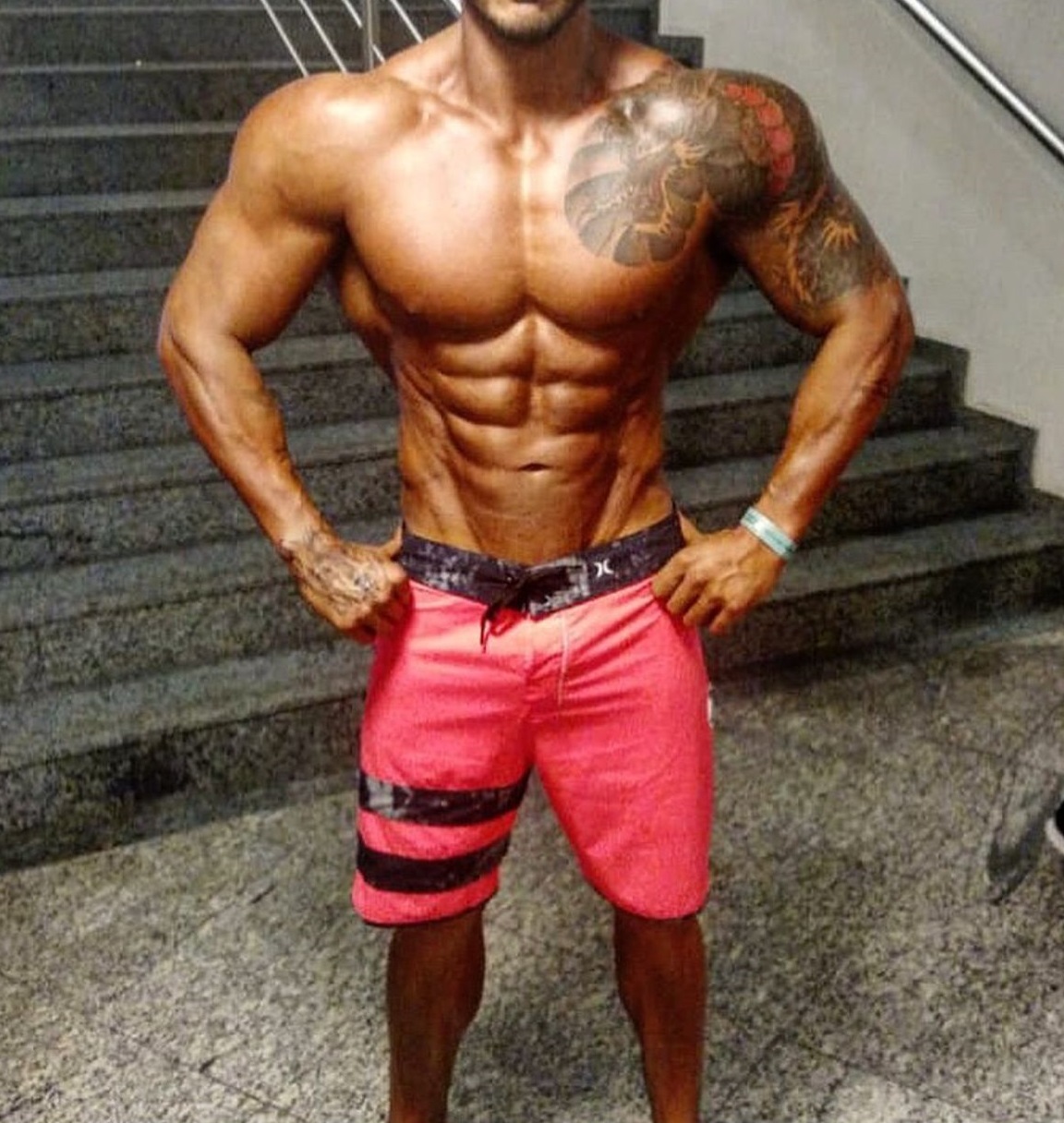

Supplement: Supplementary file 1 [file sports-10-00023-s001.zip › Supplementary Material Figure S1.jpeg]
